# A Case of Severe Neutropenia From Short-Term Exposure to Moxifloxacin

**DOI:** 10.1177/2324709617700648

**Published:** 2017-03-28

**Authors:** Weihan Chen, Peter Noel Van Buren

**Affiliations:** 1 University of Texas Southwestern Medical Center, Dallas, TX, USA

**Keywords:** moxifloxacin, fluoroquinolone, neutropenia, leucopenia

## Abstract

Moxifloxacin is commonly prescribed in the inpatient and outpatient management of community-acquired pneumonia and other common infections. We report a case of a 76-year-old man who developed severe neutropenia after several days of treatment for community-acquired pneumonia. The patient had a history of alcohol abuse; however, there were no other offending medications prescribed, and a thorough laboratory workup for other possible causes of neutropenia was negative. The patient’s neutrophils and white blood count responded quickly to cessation of fluoroquinolones. This case highlights the importance of identifying patients that might be at high risk for neutropenia that may need closer monitoring on this commonly prescribed medication.

## Case Report

A 76-year-old Hispanic male with a history of alcohol abuse, homelessness, and remote history of multiple stab wounds in the 1980s was brought to the hospital by friends due to fever, increased lethargy, and cough for the past 4 days. The cough was associated with bilateral upper abdominal pain. The cough was nonproductive other than occasional blood-tinged sputum. He had not experienced any other abnormal bleeding or bruising, but he did have general body weakness. Collateral information obtained from the patient’s friend was significant for general chronic “deterioration” over the past several months.

There was no additional medical history. The patient had undergone an exploratory laparotomy approximately 20 years prior after being stabbed in the chest and abdomen. He was unaware of any significant family medical history. His social history was significant for heavy alcohol use. He drank 1 to 2 extra-large beers (approximately 24 ounces each) daily for numerous years. He had a 40-pack year smoking history and was currently smoking 10 cigarettes per day. He denied using any illegal drugs. He was currently homeless and unemployed. He was not taking any medications, and he denied any drug allergies.

On initial physical examination, the patient was tachycardic with a pulse of 109 beats per minute. He was febrile with a temperature of 101.6°F. He was normotensive (blood pressure 138/65 mm Hg), and his pulse oximetry showed 95% on room air.

He was alert and oriented ×3. He had dry mucous membranes, but his head exam was otherwise unremarkable. He had distant heart sounds, and his lungs were clear to auscultation bilaterally. He had a midline surgical scar in the abdomen and mild tenderness to palpation in his bilateral upper extremities. His neurologic exam was unremarkable.

His initial labs were significant for a serum sodium of 128 mmol/L and serum potassium of 3.5 mmol/L. His white blood cell count (WBC) was 6.31 with a hemoglobin of 7.9, minimal corpuscular volume of 119.6, and platelet count of 158. The rest of his presenting labs are shown in Table 1. His initial chest X-ray showed no active pulmonary process or cardiac abnormality.

**Table 1.** Admission Laboratory Results.

Basic metabolic panel	
Sodium	128 mmol/L
Potassium	3.5 mmol/L
Chloride	90 mmol/L
CO_2_	22 mmol/L
Glucose	121 mg/dL
BUN	9 mg/dL
Creatinine	0.78 mg/dL
Complete blood count	
WBC	6.31 × 10^9^/L
RBC	1.89 × 10^12^/L
Hemoglobin	7.9 g/dL
Hematocrit	22.6%
Mean corpuscular volume	119.6 fL
Platelets	158 × 10^9^/L
Liver function tests	
AST	45 units/L
ALT	22 units/L
Alkaline phosphatase	42 units/L
Total bilirubin	0.4 mg/dL
Lipase	20 units/L
Urinalysis	
Specific gravity	1.016
Glucose	Negative
Ketones	Trace
Blood	Small
Nitrite	Negative
Leukocyte esterase	Negative
pH	5.5
Protein	30 mg/dL
RBC	2/HPF
WBC	<1/HPF
Bacteria	None
Squamous epithelium	1/HPF
Hyaline casts	3/HPF
Urine chemistry	
Creatinine	29 mg/dL
Osmolality	275 mOsm/kg
Potassium	31 mmol/L
Sodium	52 mmol/L

Abbreviations: BUN, blood urea nitrogen; WBC, white blood cell; RBC, red blood cell; AST, aspartate transaminase; ALT, alanine transaminase; HPF, high-power field.

**Figure 1.**
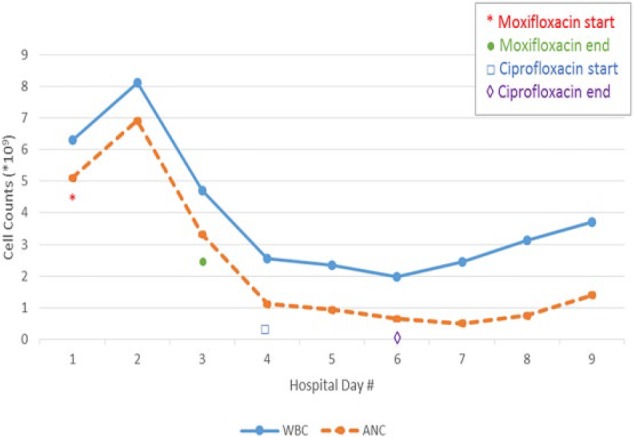
The temporal change in the white blood cell count (WBC) and absolute neutrophil count (ANC) during the patient’s hospitalization. Moxifloxacin was started on hospital day 1 (red asterisk) when both the WBC and ANC were normal. By day 3, there had been a large decrease in both WBC and ANC, prompting a change in antibiotics to ciprofloxacin and azithromycin the following day. There was continued slow decrease in the WBC and ANC, and all antibiotics were stopped on hospital day 6. The following day, and each day after there was continued increase in the WBC and ANC.

The patient was admitted and started on moxifloxacin 400 mg daily for empiric community-acquired pneumonia coverage given his clinical presentation. Due to his homelessness and general deterioration for several months, he was placed on respiratory isolation to rule out tuberculosis. Urine electrolytes and osmolarity were ordered to work up his hyponatremia, and he was empirically given normal saline because his clinical presentation was consistent with hypovolemic hyponatremia. Urine *Legionella* was also ordered. His megaloblastic anemia was thought to be secondary to alcohol use and folate/vitamin B_12_ deficiency. He was given thiamine, folate, and started on vitamin B_12_. On the first night of admission, a repeat chest X-ray was performed after his oxygen saturation decreased, which revealed a likely infiltrate in the right lung. Over the next 48 hours, he made dramatic clinical improvement remaining afebrile and with reduced cough. He remained in the hospital to continue tuberculosis rule out. The patient had daily WBC and basic metabolic profile checks. Following 2 negative acid-fast bacilli stains, it was noted that his WBC decreased to 2500 with an absolute neutrophil count (ANC) of 1200 (Figure 1). At that point, moxifloxacin was discontinued, and ciprofloxacin and azithromycin were started the next day.

The patient’s ANC continued to decline, and all antibiotics were discontinued on hospital day 6. He was placed on neutropenic precautions, and a more thorough laboratory workup for his neutropenia was undertaken, including HIV, acid-fast bacilli, influenza, viral panels, *Legionella*, hepatitis B/C, cytomegalovirus, and B_12_, which were all negative. The patient’s ANC reached a nadir of 0.51 on hospital day 7, and began to recover afterwards, increasing to 0.76 and then 1.39 before discharge. A follow-up appointment was scheduled for the patient a week later, but only a hemoglobin was drawn at that time for unclear reasons. He presented to the emergency room 3 months later with back pain (no labs drawn at that time), but was a no show for his primary care appointment the following week.

## Discussion

The patient’s presentation was consistent with moxifloxacin-induced leukopenia. He presented with a normal WBC count, although there was a notable macrocytic anemia. Nearly 24 hours after his first dose of moxifloxacin, his WBC decreased by nearly 50% and continued to decrease each day until the moxifloxacin was stopped. There was continued leukopenia following exposure to ciprofloxacin and azithromycin, and these were discontinued with a subsequent recovery of his WBC count. Other than the antibiotics, there were no offending medications prescribed to explain his leukopenia. His hemoglobin remained stable during this time with only a mild decrease in platelet count (nadir of 119 on hospital day 2).

Fluoroquinolone is an important class of broad-spectrum antibiotics used for treatment of serious bacterial infections including those of respiratory and urinary nature. Fluoroquinolones have been found to be generally well tolerated and safe. The most common adverse events include gastrointestinal and central nervous system reactions; nephrotoxicity and tendinitis are infrequent, and agents differ in phototoxicity and QTc prolongation.^1-3^ Fluoroquinolone-induced neutropenia is not well-documented; however, there are case reports of certain quinolones causing neutropenia. Chang et al reported the first case of moxifloxacin-induced neutropenia in an elderly cirrhotic woman being treated for cellulitis. Their workup was negative for bacterial or viral pathogens. The only changes made to medications was discontinuation of moxifloxacin, which led to prompt recovery of WBC.^4^ Berk et al detailed another case of moxifloxacin-induced neutropenia in a 32-year-old woman with metastatic breast cancer in remission with lobar pneumonia. Again, viral/fungal infection were excluded, and neutropenia resolved with discontinuation of moxifloxacin.^5^

Similar to the previous case reports, our patient’s workup for other causes of neutropenia was negative, and his neutropenia resolved with discontinuation of antibiotics. However, in our patient, we first tried changing antibiotics to ciprofloxacin, which continued the patient’s neutropenia. This, in addition to a case of neutropenia possibly caused by norfloxacin, provides support that neutropenia^6^ is not unique to moxifloxacin, but may be an adverse effect of the fluoroquinolone antibiotic class.

There are several mechanisms of drug-induced myelosuppression: immunologic destruction of neutrophils or direct toxicity of bone marrow precursors. Some drugs such as penicillin act as haptens to induce antibody formation against neutrophils; drugs like clozapine form toxic metabolites that deplete glutathione, which leads to increased oxidative stress and apoptosis; propylthiouracil causes an ANCA-associated lysis of neutrophils; beta-lactams, carbamazepine, and valproic acid are associated with dose-dependent inhibition of granulopoesis; ticlopidine, busulfan, methotrexate, methimazole, doxorubicin, cyclophosphamide, and chlorpromazine are associated with direct toxicity of myeloid precursors.^7^ Although no specific research has been done on the exact mechanism of fluoroquinolone-induced neutropenia, an in vitro study showed low doses of ciprofloxacin caused inhibition of hematopoietic progenitor cell proliferation.^8^ However, in our patient, and that of previous case studies, the rapid recovery of neutropenia on discontinuation of fluoroquinolones suggests that this mechanism is likely not the major pathway.

The rapid development and recovery of neutropenia suggest stable marrow production in the context of peripheral destruction of neutrophils. Hypersensitivity reactions have been described with fluoroquinolones. An immediate type 1 reaction was not likely given the absence of systemic symptoms consistent with an IgE-mediated response. Fluoroquinolones have also been reported to be associated with a delayed T-cell–mediated hypersensitivity, although the absence of the commonly seen macular papular exanthem through either drug-specific or cross-reacting mechanisms, does not support this.^9,10^ We are unaware if the patient had any prior exposure to fluoroquinolones that might have prompted an antibody-mediated response, but this mechanism remains possible. Immunoassays done by Patoia et al showed high titers of leukoagglutinins in a patient that developed neutropenia after norfloxacin administration.^6^ This supports possible ANCA or other antibody formation against neutrophils, which we also cannot exclude in our case.

Limitations of our case report include the lack of bone marrow biopsy or immunoassays to more accurately identify the mechanism of neutropenia. Additionally, because this was the patient’s first visit in our hospital system, and there was no information regarding the patient’s baseline.

## Conclusion

Our patient presented with acute leukopenia and severe neutropenia that was almost certainly related to moxifloxacin. His unique risk factor for myelosuppression include chronic alcoholism and vitamin deficiencies. A comprehensive workup was negative for any further etiology of his leukopenia. While moxifloxacin and fluoroquinolones remain important therapies for many common infections, it is necessary to monitor WBC in patients at high risk for myelosuppression. We report a case of moxifloxacin with leukopenia, and we introduce the idea that neutropenia may be an adverse effect of the fluoroquinolone class of antibiotics. Although this adverse reaction is rare, it needs to be considered in patients who have significant drops in ANC after starting fluoroquinolones, as discontinuation has led to prompt recovery of WBC and ANC in all case reports.
